# Impact of COVID-19 and lockdowns on pulmonary embolism in hospitalized patients in France: a nationwide study

**DOI:** 10.1186/s12931-021-01887-6

**Published:** 2021-11-20

**Authors:** Pierre Tankere, Jonathan Cottenet, Pascale Tubert-Bitter, Anne-Sophie Mariet, Guillaume Beltramo, Jacques Cadranel, Lionel Piroth, Philippe Bonniaud, Catherine Quantin

**Affiliations:** 1 Reference Center for Rare Pulmonary Diseases, Pulmonary Medicine and Intensive Care Unit Department, Dijon University Hospital, BP 77908, 21079 Dijon, France; 2 CHU de Dijon - Service de Biostatistique et d’Informatique Médicale, BP 77908, 21079 Dijon CEDEX, France; 3 University of Burgundy and Franche-Comté, Dijon, France; 4 Université Paris-Saclay, UVSQ, Univ. Paris-Sud, Inserm, High-Dimensional Biostatistics for Drug Safety and Genomics, CESP, Villejuif, France; 5 Clinical Investigation Center, Clinical Epidemiology/Clinical Trials Unit, Inserm, CIC 1432, Dijon, France ; Dijon University Hospital, Dijon, France; 6 Chest Department and Constitutive Center for Rare Pulmonary Disease, Hôpital Tenon, AP-HP, Inflammation-Immunopathology-Biotherapy Department (DHU i2B) and Sorbonne Université, 75020 Paris, France; 7 Faculty of Medicine, University of Burgundy and Franche-Comté, Dijon, France; 8 Infectious Diseases Department, Dijon University Hospital, BP 77908, 21079 Dijon, France; 9 INSERM, LNC UMR1231, LipSTIC LabEx Team, Dijon, France

**Keywords:** COVID-19, Pulmonary embolism, Medico-administrative data, Hospital, Lockdown, SARS-COV2

## Abstract

**Background:**

This study assessed the impact of the COVID-19 epidemic on overall hospitalizations for pulmonary embolism (PE) in France in comparison with previous years, and by COVID-19 and non-COVID-19 status.

**Methods:**

Hospitalization data (2017–2020) were extracted from the French National Discharge database (all public and private hospitals).

We included all patients older than 18 years hospitalized during the 3 years and extracted PE status and COVID-19 status (from March 2020). Age, sex and risk factors for PE (such as obesity, cancer) were identified. We also extracted transfer to an intensive care unit (ICU) and hospital death. The number of PE and the frequency of death in patients in 2019 and 2020 were described by month and by COVID-19 status. Logistic regressions were performed to identify the role of COVID-19 among other risk factors for PE in hospitalized patients.

**Results:**

The overall number of patients hospitalized with PE increased by about 16% in 2020 compared with 2019, and mortality also increased to 10.3% (+ 1.2%). These increases were mostly linked to COVID-19 waves, which were associated with PE hospitalization in COVID-19 patients (PE frequency was 3.7%; 2.8% in non-ICU and 8.8% in ICU). The final PE odds ratio for COVID-19 hospitalized patients was 4 compared with other hospitalized patients in 2020.

The analyses of PE in non-COVID-19 patients showed a 2.7% increase in 2020 compared with the previous three years.

**Conclusion:**

In 2020, the overall number of patients hospitalized with PE in France increased compared to the previous three years despite a considerable decrease in scheduled hospitalizations. Nevertheless, proactive public policy focused on the prevention of PE in all patients should be encouraged.

**Supplementary Information:**

The online version contains supplementary material available at 10.1186/s12931-021-01887-6.

## Background

The global severe acute respiratory syndrome coronavirus 2 (SARS-Cov2) pandemic was declared on March 11, 2020. One year later, more than 120 million cases of coronavirus disease-2019 (COVID-19) and more than 2.8 million COVID-19-related deaths have been reported according to the World Health Organization. In France, the COVID-19 epidemic has caused more than 110,000 deaths, 83,000 of which were in hospital. While SARS-Cov2 primarily infects respiratory epithelial cells, it also induces endothelial dysfunction. Soon after the outbreak, it was identified that COVID-19 was associated with an hypercoagulable state [1] that was linked to an increased prevalence of pulmonary embolism (PE). Even though the exact interplay between COVID-19 and PE has not been definitely established, numerous papers [2, 3] have described an endothelial effect and an inflammatory burden associated with COVID-19, which contributes to venous thrombosis.

The frequency of PE was found to reach 3.5% in hospitalized patients with COVID-19 and 13.7% in those admitted to the intensive care unit (ICU) [4]. This discovery led many countries to recommend the use of chest computed tomography scans with pulmonary angiogram (CTPA) in patients with severe COVID-19 and disproportionate hypoxemia or sudden clinical deterioration, and sometimes systematically in patients with blood D-Dimers > 3 000 µg/L [5]. In addition, enhanced thromboprophylaxis was prescribed for hospitalized patients with body mass index > 30 kg/m^2^ or on high flow oxygen therapy or mechanical ventilation [6, 7].

Lockdowns and restrictions of varying durations and intensities were implemented all around the world in an effort to control the pandemic. In France, the first wave of COVID-19 started on February 23, 2020, leading to an initial two-month lockdown from March 17 to May 11, 2020, with the peak of the epidemic in mid-April 2020. A second and less stringent one-month lockdown was implemented from October 28 to November 28, 2020. These lockdown periods caused a drastic backlog in scheduled hospital stays for non-COVID-19 patients, especially for surgical interventions [8]*.* The first COVID-19 lockdown also significantly decreased the number of hospitalizations for trauma linked to traffic accidents [9] and for severe diseases (e.g. strokes [10] and myocardial infarctions [11].

Few studies have directly compared the impact of COVID-19 and/or the lockdown on the incidence of PE. As far as we know, only two studies have been conducted on nationwide data, one in Denmark [12] and one in the UK [13]. They reported divergent trends, with the Danish study finding a decline in PE hospitalizations during COVID-19, whereas the UK study showed an increase in PE. However, these studies focused only on the first wave of COVID-19 in 2020.

Our main objective was to assess whether the COVID-19 pandemic in France was associated with a change in PE hospitalizations in patients with and without COVID-19. We also aimed to study the impact on PE hospitalizations in 2020 as a whole, with the two waves of COVID-19 and the associated lockdown. We hypothesized that the reductions in physical activity associated with the lockdowns may have led to an increase in PE hospitalizations, surpassing the previous increase in PE. We also hypothesized that enhanced anticoagulation policy as well as adaptation to lockdown restrictions may have led to a different effect on PE hospitalizations between the lockdowns for the first and second waves.

## Methods

The study design was a retrospective cohort study using the national hospital database (*Programme de Médicalisation des Systèmes d’Information*, PMSI), which is designed to include discharge summaries for all inpatients admitted to public and private hospitals in France. The information in these abstracts is anonymous and covers both medical and administrative data. Diagnoses identified during the hospital stay are coded according to the 10th edition of the International Classification of Diseases (ICD-10), and procedures performed during the hospitalization are coded according to the French Common Classification of Medical Procedures.

Inclusion criteria were all patients older than 18 years hospitalized in France from January 1st 2017 to December 31st 2020.

Outcomes for each hospitalization were binary variable of PE diagnosis and diagnosis of COVID-19. Hospital stays for COVID-19 were identified by the primary diagnoses (PD), related diagnoses (RD) or associated diagnoses (AD) by the ICD-10 codes U0710, U0711, U0712, U0714 or U0715, used in previous studies [10, 14]. From March 1, 2020, we separated patients according to whether COVID-19 was recorded on their discharge abstract. PE diagnosis were identified by ICD-10 code I26. Considering that most COVID-19 patients had their first stay in the emergency department or short term COVID-19 unit, we considered that PE was present on admission if it was diagnosed in the first hospital unit of stay.

We also created a COVID-19 sub-cohort including all patients older than 18 years hospitalized for COVID-19, with and without PE, admitted after March 1, 2020, and discharged before December 31, 2020.

The variables and risk factors extracted for each inpatient stay were: age, sex, transfer to ICU, and hospital death. We identified known risk factors for PE, such as obesity and cancer (including hematologic and solid tumors), identified through the abovementioned ICD-10 codes (PD, RD or AD) in the discharge abstracts for the included hospital stays**.** We also identified any surgery in the two months preceding the stay with COVID-19 or PE. Finally, we were able to identify postpartum stays for women who gave birth in 2019 and 2020.

The statistical analyses of data were by descriptive methods, using tabulations of summary statistics and distributions over time that were also presented graphically with relevant categorizations, as detailed below. Qualitative variables are summarized as frequencies (percentages), and quantitative variables are summarized as means ± standard deviations (SD) and medians [interquartile range (Q1–Q3)].

The number of patients hospitalized with PE and the frequency of PE among all adults (18 years or older) hospitalized per year were determined.

The characteristics of hospitalized patients with PE were described by summary statistics. The different variables analyzed in the cohort of patients hospitalized for PE were compared using the Chi-2 test (for qualitative variables) and Kruskal–Wallis test or Mann–Whitney test (for quantitative variables), according to the year and the COVID-19 status.

The number of patients hospitalized with PE and the death frequency for patients with PE for 2019 and 2020 were tabulated by month from March to December. The distribution of monthly admission for PE was also presented graphically.

In the COVID-19 cohort, we calculated the overall frequency of PE from March to December and then separately for patients admitted to the ICU. The distributions over time were presented graphically. We then estimated the risk of PE among all patients hospitalized between March and December 2020, using logistic regressions (Additional file 1: Table S1). These regressions, which aim to study the effect of COVID-19 on the risk of PE, were adjusted for age (18–50, 51–70 and > 70), sex, obesity, and cancer. The variables included in the multivariate models were those that were significant in univariate analysis, with a p-value < 0.20. Correlations were studied and interactions tested. The results were reported as odds ratios (OR) and 95% confidence intervals (CI).

The statistical significance threshold was set at < 0·05. All analyses were performed using SAS (SAS Institute Inc, Version 9.4, Cary, NC).

This study was approved by the Ethics and Scientific Committee for Research, Studies and Evaluation in Health (*Comité Ethique et Scientifique pour les recherches, les études et les évaluations dans le domaine de la santé,* CESREES, June 9, 2020) and the French Institute of Health Data (*Institut National des Données de Santé*, INDS, registration number 1611357, June 15, 2020) and authorized by the French Data Protection Authority (*Commission Nationale de l’Informatique et des Libertés*, CNIL, registration number DR-2020–250, July 3 2020).

## Results

**Fig. 1:**
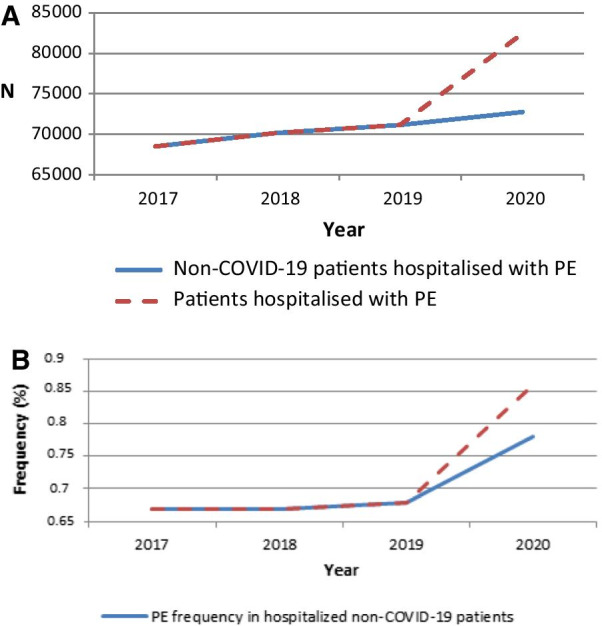
Patients hospitalized with pulmonary embolism (2017–2020) (**A**) and yearly frequencies of PE in hospitalized patients (**B**)

The number of adult patients hospitalized for all causes in French public and private hospitals decreased by 9.4% in 2020 compared with 2019 (approximately 10.5 million in 2019 and 9.5 million in 2020, see Table 1). The reduction in 2020 was of almost 12% when COVID-19-related hospitalizations (approximately 260 000) were excluded. In contrast, patients hospitalized with PE increased from 2019 to 2020 by about 16% (+ 11,000, Table 1 and Fig. 1A). This increase also contrasts with the relative stability observed before 2019 (e.g. 1.4% increase between 2018 and 2019).

**Table 1:** Patients hospitalized with pulmonary embolism in France: Summary statistics (2017–2020)

	2017	2018	2019	2020	p-value* (comparison of the 4 years)	2020 COVID-19 from 1st March to 31st December	p-value** (comparison to 2019)	2020 non-COVID-19 *from 1*^*st*^* March to 31*^*st*^* December*	p-value** (comparison to 2019)
Hospitalized patients with PE (n)	68 520	70 201	71 167	82 448		9 693		59 848	
Rate of change (%)		2.5%	1.4%	15.8%					
Hospitalized patients (n)	10 297 403	10 417 988	10 527 664	9 538 270		259 482		7 717 759	
PE frequency among hospitalized patients (%)	0.67% [0.665–0.675]	0.67% [0.665–0.675]	0.68% [0.675–0.685]	0.86% [0.854–0.866]		3.74% [3.67–3.81]		0.77% [0.76–0.78]	
Age (years)									
Mean ± sd	70 ± 16	70 ± 16	69 ± 16	69 ± 16	< 0.01	69 ± 16	< 0.01	69 ± 17	0.0033
Median [Q1-Q3]	72 [60–82]	71 [60–82]	72 [60–82]	71 [59–82]	0.0007	70 [59–81]	< 0.01	72 [59–82]	0.5491
Min–Max	18–109	18–105	18–109	18–108		18–104		18–108	
Female frequency in hospitalized PE	51.6% [51.2–52.0]	52.3% [51.9–52.7]	51.6% [51.2–52.0]	49.8% [49.5–50.1]	< 0.01	40.6% [39.6–41.6]	< 0.01	50.8% [50.4–51.2]	0.0023
Obesity frequency in hospitalized PE	8.6% [8.4–8.8]	8.7% [8.5–8.9]	8.8% [8.6–9.0]	9.5% [9.3–9.7]	< 0.01	13.1% [12.4–13.8]	< 0.01	9.0% [8.8–9.2]	0.1656
Cancer frequency in hospitalized PE	23.1% [22.8–23.4]	22.7% [22.4–23.0]	23.2% [22.4–23.0]	21.5% [21.2–21.8]	< 0.01	10.2% [9 .6–10.8]	< 0.01	22.9% [22.6–23.2]	0.1127
Surgery frequency in the 2 months before hospitalization with PE	NA	NA	5.2% [5.0–5.4]	4.6% [4.5–4.7]	NA	2.7% [2.4–3.0]	< 0.01	5.2% [5.0–5.4]	0.9707
Initial*** PE in hospitalized PE	85.2% [84.9–85.5]	85.5% [85.2–85.8]	82.0% [81.7–82.3]	81.8% [81.5–82.1]	< 0.01	75.5% [74.6–76.4]	< 0.01	82.3% [82.0–82.6]	0.1546
ICU frequency in hospitalized PE	25.3% [25.0–25.6]	25.0% [24.7–25.3]	25.2% [24.9–25.5]	25.8% [25.5–26.1]	0.0043	34.3% [33.4–35.2]	< 0.01	24.8% [24.5–25.1]	0.0960
Death frequency among hospitalized PE	9.2% [9.0–9.4]	9.0% [8.8–9.2]	9.1% [8.9–9.3]	10.3% [10.1–10.5]	< 0.01	19.3% [18.5–20.1]	< 0.01	9.1% [8.9–9.3]	0.8510

*PE*: pulmonary embolism, *SD* Standard deviation, *ICU* intensive care unit, *NA* not applicable

* Kruskal–Wallis test and Brown-Mood test for comparison of age and Chi-2 test for other variables

**Mann–Whitney test and Brown-Mood test for comparison of age and Chi-2 test for other variables

*** PE diagnosed in the first unit of hospitalization

Among all hospitalized patients, the frequency of PE was stable in 2017, 2018 and 2019 (0.67%, 0.67% and 0.68% of patients respectively), but the frequency increased to 0.86% in 2020. Regarding non-COVID-19 patients, the frequency of PE among hospitalized patients increased to 0.77% in 2020. The marked impact of COVID-19 in 2020 versus the previous years can be seen clearly in Fig. 1A and B and in Table 1 (3.7% of PE among patients hospitalized with COVID-19). When the analysis was limited to March to December, the increase in patients hospitalized with PE for non-COVID-19 patients in 2020 was 2.7% when compared to the corresponding period in 2019. It should be noted that in 75% of COVID-19 hospitalized patients with PE, the condition was diagnosed in the first unit of hospitalization (Table 1).

**Fig. 2:**
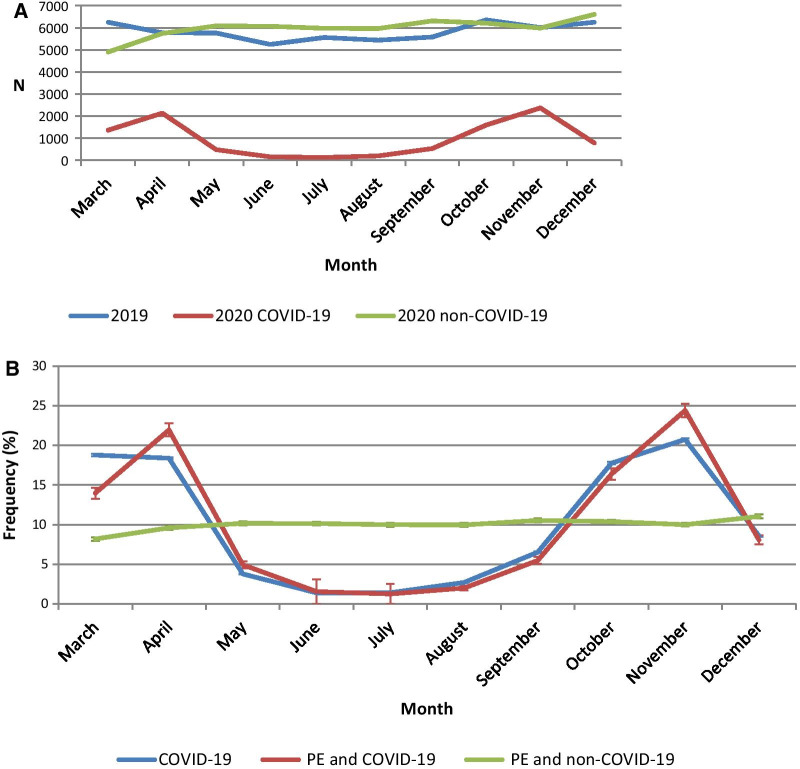
Patients hospitalized with pulmonary embolism per month. **A** Numbers of patients hospitalized with pulmonary embolism per month with a comparison between 2019 and 2020. **B** Monthly distribution (%) of patients hospitalized with COVID-19 or pulmonary embolism in 2020 (with COVID-19 or not) including error bars (error bars were calculated for each month but are not necessarily easily readable because the range of confidence intervals is small)

**Fig. 3:**
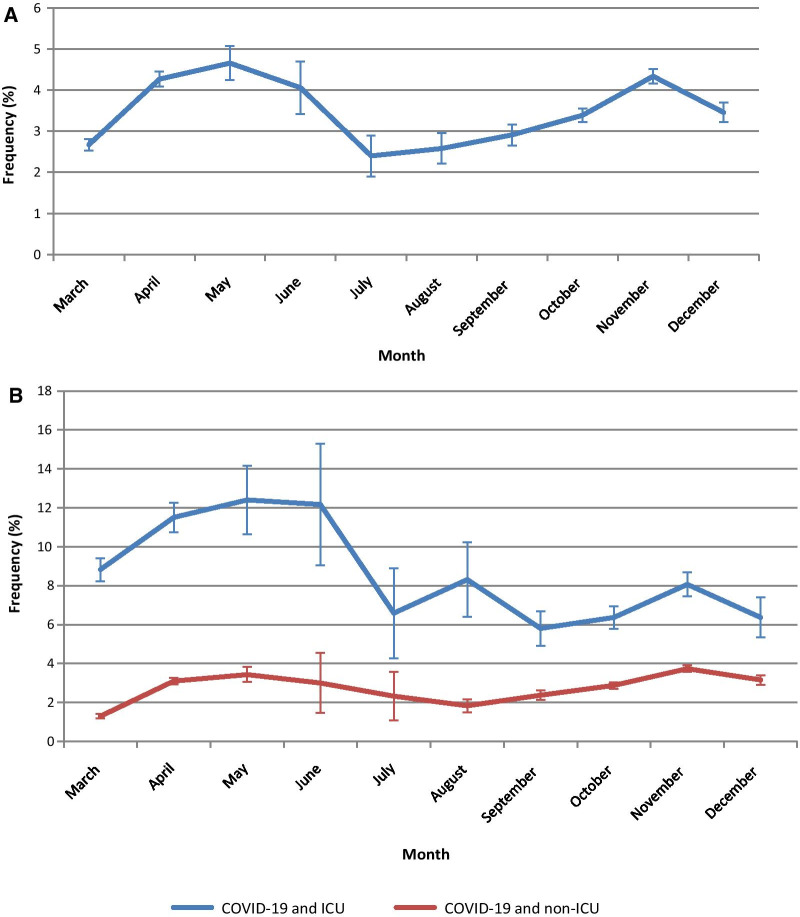
Monthly frequencies of pulmonary embolism in hospitalized COVID patients including error bars (error bars were calculated for each month but are not necessarily easily readable because the range of confidence intervals is small): globally for all COVID-19 patients (**a**) and separately for patients hospitalized in ICU or not (**b**)

On a monthly basis, there was an increase in the total number of patients hospitalized with PE in 2020 compared to 2019 in each month (except for March 2020), regardless of the waves of the pandemic and the lockdowns (see Fig. 2 and Additional file 1: Figure S1). This was also observed for non-COVID-19 patients. After an initial downturn in non-COVID-19 patients in March (21% decrease in 2020 compared with 2019), rapid increases were systematically observed the following months compared with 2019. This phenomenon was particularly visible during the first wave and to a lesser extent during the second wave (Additional file 1: Figure S1). In hospitalized COVID-19 patients, peaks in the frequency of PE corresponded to the two COVID-19 waves in the pre- and post- summer months (see Figs. 2 and 3). The average over 5 months seems to suggest an overall downward trend in the frequency of patients hospitalized for COVID-19 with PE (Additional file 1: Figure S2).

For hospitalized patients with PE (see Table 1), mean age (70 years) was stable over the years and did not differ among COVID-19 and non-COVID-19 patients hospitalized in 2020. Whereas sex ratio was 1:1 from 2017 to 2019 overall, men were more frequent among COVID-19 patients with PE (59.4%). Obesity was also more frequent in COVID-19 patients as opposed to non-COVID-19 patients (13% vs 9%). Underlying cancer was less frequent in COVID-19 than in non-COVID-19 hospitalized patients (10.2% vs 22.9%), as well as recent surgery (2.7% vs 5.2%).

Whereas the frequency of admission in ICU of hospitalized patients with PE was stable before 2020 (around 25%), patients hospitalized with PE are more frequently admitted to ICU in COVID-19 than non-COVID-19 patients (34% vs 25%) in 2020 (Table 1). The PE frequency is higher in COVID-19 patients hospitalized in ICU (8.8%) than in non-ICU (2.8%). Figure 3 shows some differences in monthly PE frequency in ICU vs non-ICU with an overall trend to decrease being more prominent in ICU over 2020.

The mortality frequency was roughly constant in patients hospitalized with PE before 2020 (9% to 9.2%). It increased to 10.3% in 2020 (relative risk (RR) of death of 1.14 [95% CI 1.10–1.17] compared to 2019), due to the high mortality among COVID-19 patients (19.3%, RR of death of 2.13 [2.03–2.23]) compared with non-COVID-19 patients (9.1%, RR of death of 1.00 [0.96–1.03]) (Table 1). Mortality frequency was 20.1% for COVID-19 patients with PE versus 15.7% in COVID-19 patients without PE (p < 0.01). This was true for both ICU and non ICU subgroups (data not shown). Our data also show a decrease in the mortality of COVID-19 with PE between the two waves; it was as high as 28% in March 2020 versus 18% to 20% in October and November 2020.

In the logistic regression estimating the risk of PE between March and December 2020, we found that COVID-19 infection was associated with PE both in univariate and multivariate analysis, with a fourfold increase in risk (aOR = 3.98 95%CI [3.90–4.07]) after adjustment for all variables cited above, in particular the factors usually associated with PE such as obesity and cancer. It should be noted that the risk factors for PE were similar in 2019 and 2020 (Additional file 1: Table S1).

Considering the year 2019 as a reference, we also found that hospitalized patients without COVID-19 in 2020 were at a higher risk of PE (aOR = 1.19 [1.18–1.21]), with the same adjustment (Additional file 1: Table S1).

## Discussion

In this French nationwide study, the total number of patients hospitalized with PE in 2020 increased by about 16% compared with 2019, and mortality in hospitalized PE patients increased to 10.3% (+ 1.2%). Thus, overall hospitalization with PE increased in France during the COVID-19 pandemic, contrary to Danish reports [15] but similar to the UK [13]. While the Danish study showed a significant decline in hospitalizations for PE during the first wave and lockdown, the magnitude of the first wave was lower in Denmark and the lockdown was shorter than in France. In the U.K on the contrary, there was an estimated adjusted relative risk of PE of 1.5 during the 5 first months of the pandemic compared to the “pre-COVID-19 period” from Feb 1, 2018, to the outbreak of the pandemic. It is worth noting that these two studies only investigated the 1st wave of the epidemic, whereas our results include the first 2 waves of the epidemic in France in 2020.

The increases in PE hospitalizations in France were mostly linked to the two COVID-19 waves, which were also associated with PE hospitalizations in COVID-19 patients. However, it is important to note that our analyses of PE in non-COVID-19 patients show that there was a 2.7% increase in 2020 compared with the previous three years. The frequency of patients hospitalized with PE needing ICU care also increased compared to previous years, but for COVID-19 patients only. Our findings relative to COVID-19 hospitalizations are consistent with official French government figures available from May 2020 onwards. There is a slight discrepancy in the peak of COVID-19, probably due to time elapsed between the first symptoms and severe stage of the disease requiring hospitalization, as described in Huang et al. (Additional file 1: Figure S1) [16].

Among hospitalized COVID-19 patients, the observed overall frequency of PE was 3.7% (2.8% in non-ICU and 8.8% in ICU). Our data suggest that 25% of COVID-19-associated PE cases were diagnosed secondarily during the hospitalization, which is much higher than the 15 to 18% observed in the years before the pandemic. It should also be underlined that COVID-19 diagnosis has the highest odds ratio among the risk factors analyzed in hospitalized PE in 2020.

The threefold higher frequency of PE in the ICU reflects that PE is a sign of severe COVID-19 and that ICU hospitalization itself increases the risk of PE. The proportion of CTPA leading to PE diagnosis in COVID-19 patients do not suggest an excessive rate of screening in ICU patients (data not shown). However, we observed lower frequencies than in several recent studies [12, 17], (2.6% to 8.9% in non-ICU and 13.7% to nearly 33% in ICU) [2, 4]. This might reflect an over-representation of the ICU population in these studies. Higher frequencies in small cohorts have already been discussed in a meta-analysis by Gallastegui et al. [18], who described frequencies similar to ours in cohorts with more than 400 patients. Our results in COVID-19-associated PE in ICU and non-ICU patients are in line with a recent meta-analysis [19]. Finally, contrary to the higher frequencies reported by Jevnikar et al. [20], our data did not result from systematic CTPA on admission and might better reflect clinically significant PE leading to CTPA indication, and linked to clinical symptoms during the whole hospital stay.

The high frequency of PE in COVID-19 patients is due mainly to COVID-19 itself and the associated inflammatory burden. However, there are some significant differences in the PE risk factors prevalent in COVID-19 patients with PE. For instance there was a higher frequency of obesity, which is is a factor related to COVID-19 disease severity, leading both to a higher risk of PE (more sedentary lifestyle) and to more likely transfer to ICU. The frequency of cancer and recent surgery were lower in COVID-19 PE than in non-COVID-19 PE patients. These lower frequencies might be indirectly linked to more preventive behaviors in patients with cancer or recent surgery, resulting in a fewer cases of COVID-19 and, ultimately, less COVID-19-associated PE. The odds ratio associated with the usual PE risk factors derived from logistic regression of hospitalized PE were very similar to 2019 (Additional file 1: Table S1).

The very modest decrease in PE frequency in COVID-19 patients between the two waves of the epidemic may appear surprising given the enhanced preventive anticoagulation policy that was associated with a reduction in inflammatory biomarkers [21]. One hypothesis could be that greater attention was paid to the risk of PE, which might have resulted in a more inclusive indication for CTPA, as already reported [22]. An overdiagnosis of PE when there were perfusion defects unrelated to PE in dual energy CT angiography is also a possible explanation [23]. The potential overdiagnosis bias should be considered alongside a potential underdiagnosis bias, which may have occurred both in non-COVID-19 patients and in less severe non-ICU COVID-19 patients. Indeed those groups might receive less medical consideration for events that are not hemodynamically significant. This seems particularly plausible in the context of the COVID-19 epidemic and the subsequent need for CTPA in severe COVID-19 patients.

Furthermore, our database shows that mortality was significantly greater for COVID-19 patients with PE versus COVID-19 patients without PE. This result contrasts with a previous study [24] which found no difference*.* This might be due to the greater power of our national data or to national differences in patient management.

As for PE in hospitalized non-COVID-19 patients, there was a surprising notable increase in patients compared to 2019. Our nationwide data illustrate a phenomenon that has been described on a local scale in Japan [25]. In contrast, studies based on the same French nationwide database did not observe this phenomenon for other vascular diseases (such as strokes [10] or myocardial infarction [11]) during the first wave of the epidemic. In fact, they found a significant and sustained decrease in hospital admissions in 2020, driven by a sharp drop in admission during the first lockdown and a lack of compensation in the post-lockdown period. The overall increase in non-COVID-19 patients hospitalized with PE observed here is not due to changes in the usual PE risk factors. Indeed, in 2020, the frequencies of obesity, cancer and recent surgery in these patients do not differ significantly from the corresponding prevalence from 2017 to 2019. However, this phenomenon might reflect a decrease in physical activity [26, 27] associated with pandemic restrictions and lockdown, and subsequent increases in the risk of thromboembolic diseases. Other potential risk factors include an increase in smoking [28] or poor adherence to usual treatment in non-COVID-19 patients receiving less medical attention. On the other hand, an increase in the use of CTPA due to the suspicion of COVID-19, even in non-COVID-19 patients, may have identified PE cases which would have been overlooked otherwise.

The lockdown had only a slight influence on the monthly distribution of non-COVID-19 hospitalized PE when compared with COVID-19 patients, and the effect appears to be smaller than the reported decreases in Denmark [15] and Austria [29]. There was, however, an early downturn followed by a rapid increase in hospitalized non-COVID-19 patients with PE which deserves attention. The population may have been reluctant to seek medical care during the early phase of the pandemic and lockdown (in March) due to public health policy and fear of contamination [30]. It could also be a delayed effect of the restrictions and lockdown. Even if the lockdowns had clear beginning and end dates, people were certainly doing less physical activity, leading to a more sedentary lifestyle. In addition, the less marked slowdown and catch-up during the second wave could indicate more effective hospital reorganization and the habituation of patients to the pandemic and lockdown, meaning that they were more inclined to seek medical care and maintain a certain level physical activity. Differences with Denmark and Austria may be explained by (1) epidemiological differences including shorter lockdowns and less dramatic COVID-19 peaks, and (2) hypothetical differences in care-seeking behaviors (more likely to consult for potential cardiac symptoms than in France).

The overall increase in PE will possibly lead to increased chronic complications of PE, such as new cases of chronic thromboembolic pulmonary hypertension (CTEPH). CTEPH is both an interesting retrospective marker of COVID-19-associated PE and a conditions that will require particular consideration when diagnosing chronic dyspnea in the near future. Unfortunately, our study does not allow us to assess or predict this risk.

Further investigations will be needed to clarify the relationship between deep vein thrombosis (DVT) and PE in COVID-19 and in comparison with other ARDS. As suggested in metanalyses on DVT and PE [24, 31], there is a major to identify the proportion of PE resulting from DVT embolism and the proportion resulting from COVID-19 pulmonary vasculopathy. The endothelial effect of ARDS, which is well-known, is more correlated with duration of disease than with a specific cause [32, 33]). In COVID-19, the endothelial effect is also attributed to non-specific mechanisms such as severe hypoxemia, which in turn predisposes to thrombosis by increasing blood viscosity, complement activation, and cytokine storms, resulting in microthrombosis [1]**.** However, it seems that antiphospholipid antibodies may also play a role in COVID-19 coagulopathy [34] as well as fibrinolysis resistance and platelet aggregation [35]**.**

This article provides a nationwide report covering all of 2020, including the first two COVID-19 waves and associated lockdowns. It distinguishes COVID-19 vs non-COVID-19 patients on a monthly basis and provides a comparison with the three previous years. This work is unique for the large number of patients included and the comprehensiveness of the data. In France, the national hospital database includes information from all private and public French hospitals; it is used for the allocation of hospital budgets and encourages high levels of data coherence, accuracy, and exhaustiveness. The accuracy of PMSI for these diagnoses has been previously tested in previous literature, for instance for obesity [36] or for EP, with a positive predictive value of 99% for PE diagnosis, indicating that these codes can be used in comparative pharmacoepidemiological studies [37]. Our study also adds valuable data to the pending debate over the best anticoagulation policy in COVID-19 hospitalized patients, and particularly the balance between safety and efficacy [38].”

Although this study includes data collected on a national level with almost 80 000 adult patients of all ages hospitalized with PE and more than 250 000 hospitalized with COVID-19, we recognize that it has several limitations. One limitation is the potential for biases related to misclassification or under-detection. However, the reliability of the detection of PE and COVID-19 in our medico-administrative database is likely very high considering its severity and the impact on patient management. Moreover, the quality and completeness of the coding are evaluated locally by the medical information departments of the individual institutions and nationally by the national health insurance system. Since the PMSI only includes data collected from hospitalized patients, we have no data from outpatients and or from patients deceased at home. For instance, our data does not allow us to provide more details about the anticoagulant treatment (prophylactic or curative) received by the patients, and further investigation by using another database which would include drugs prescribed in hospital or in an ambulatory setting would be very interesting. There are also some limits due to the study design, for instance we cannot guarantee standardized CTPA screening policy and the PMSI does not provide data to study the method use to diagnose PE. For some variables, information may not have been completed in the discharge abstract when there was no direct impact on patient care during hospitalization (e.g. tobacco use). Nevertheless, this is most often the case for day hospitalizations, and is less likely to occur in the data for inpatient hospitalizations considered in this study.

Finally, PE during pregnancy could not be taken into account seeing as data for 2021 were not yet available to identify deliveries. However, we were able to identify postpartum stays for women who gave birth in 2019 and 2020. It was not possible to take into account the effect of surgery in the logistic regression either since surgical activity was heavily affected in this very particular context, in particular during the lockdown periods.

In conclusion, the overall number of patients hospitalized with PE in France increased in 2020 compared with the previous three years, and not only because of COVID-19. The increase was visible throughout the studied months of the COVID-19 pandemic, despite a sharp decrease in planned surgeries during the lockdowns. This unique nationwide database shows a high frequency of PE among COVID-19 hospitalized patients, thus confirming the results obtained in some meta-analyses with smaller cohorts. In addition, it shows a higher mortality rate among COVID-19 patients with PE compared to those without PE, which adds information to previous reports. This study also indicates that there was an increase in PE in non-COVID-19 patients, particularly during the waves of COVID-19 and associated lockdowns.

Our results suggest that there is a need for proactive public policy concerning PE prevention in both COVID-19 and non-COVID-19 patients. This could include the promotion of physical activity as well as medical awareness, which would imply drawing even more attention to the burden PE. The apparent modest efficacy of treatments and anticoagulation approaches on PE in COVID-19 is also another strong rational for the importance of extensive SARS-COV2 vaccination.

## Supplementary Information

**Additional file 1.** **Supplementary Figure 1:** 1A) relative changes (%) between 2019 and 2020 in the number of patients hospitalized with pulmonary embolism overall and in non-COVID-19 patients. 2B) monthly distribution (%) of patients hospitalized with pulmonary embolism in 2019 and 2020 (with COVID or not). **Supplementary Figure 2:** pulmonary embolism frequency in hospitalized COVID-19: 5-month floating average. **Supplementary Table 1:** logistic regression to study the effect of COVID-19 on the risk of pulmonary embolism among all hospitalized patients. **Supplementary Figure 3:** monthly distribution (%) of patients hospitalized with COVID-19 or pulmonary embolism in 2020 (with COVID-19 or not) and respective incidence of COVID-19 in France (per 100,000 persons) derived from official French Government figures available from May 2020.

## Data Availability

The PMSI database was transmitted by the national agency for the management of hospitalization data. The use of these data by our department was approved by the National Committee for data protection. We are not allowed to transmit these data. PMSI data are available for researchers who meet the criteria for access to these French confidential data (this access is submitted to the approval of the National Committee for data protection) from the national agency for the management of hospitalization (ATIH—Agence technique de l'information sur l'hospitalisation). Address: Agence technique de l'information sur l'hospitalisation, 117 boulevard Marius Vivier Merle, 69,329 Lyon Cedex 03.
