# Supplementary material for: Impact of COVID-19 and lockdowns on pulmonary embolism in hospitalized patients in France: a nationwide study
Source: Respir Res. 2021 Nov 20;22:298. doi: 10.1186/s12931-021-01887-6 (PMC8605779; doi:10.1186/s12931-021-01887-6)
Supplement: Supplementary file 1 — Additional file 1. Supplementary Figure 1: 1A) relative changes (%) between 2019 and 2020 in the number of patients hospitalized with pulmonary embolism overall and in non-COVID-19 patients. 2B) monthly distribution (%) of patients hospitalized with pulmonary embolism in 2019 and 2020 (with COVID or not). Supplementary Figure 2: pulmonary embolism frequency in hospitalized COVID-19: 5-month floating average. Supplementary Table 1: logistic regression to study the effect of COVID-19 on the risk of pulmonary embolism among all hospitalized patients. Supplementary Figure 3: monthly distribution (%) of patients hospitalized with COVID-19 or pulmonary embolism in 2020 (with COVID-19 or not) and respective incidence of COVID-19 in France (per 100,000 persons) derived from official French Government figures available from May 2020. [file 12931_2021_1887_MOESM1_ESM.docx]

**Additional Material**

**Additional file 1: Figure S1: 1A) relative changes (%) between 2019 and 2020 in the number of patients hospitalized with pulmonary embolism overall and in non-COVID-19 patients. 2B) monthly distribution (%) of patients hospitalized with pulmonary embolism in 2019 and 2020 (with COVID or not) including error bars (error bars were calculated for each month but are not necessarily easily readable because the range of confidence intervals is small)**

**Additional file 1: Figure S2: pulmonary embolism frequency in hospitalized COVID-19 patients including error bars (error bars were calculated for each month but are not necessarily easily readable because the range of confidence intervals is small): 5-month floating average**


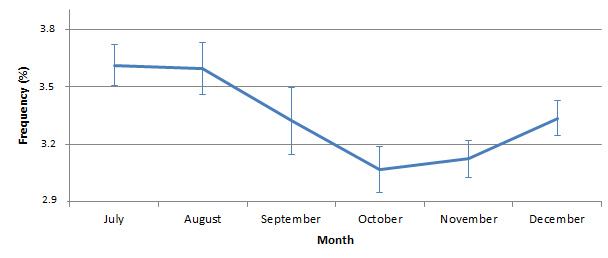


**Additional file 1: Table S1: logistic regression to study the effect of COVID-19 on the risk of pulmonary embolism among all hospitalized patients**

|  | 2019 | | 2020 | |  | 2019-2020 | |
| --- | --- | --- | --- | --- | --- | --- | --- |
|  | Crude OR [95% CI] | aOR [95% CI] | Crude OR [95% CI] | aOR [95% CI] |  | Crude OR [95% CI] | aOR [95% CI] |
| COVID-19 | - | - | 5.01 [4.90-5.12] | 3.98 [3.90-4.07] | Year (ref=2019) |  |  |
|  |  |  |  |  | 2020 without COVID-19 | 1.21 [1.20-1.22] | 1,19 [1,18-1,21] |
|  |  |  |  |  | 2020 with COVID-19 | 6.05 [5.92-6.18] | 4,73 [4,63-4,84] |
| Obesity | 2.21 [2.15-2.27] | 2.56 [2.49-2.62] | 2.36 [2.30-2.42] | 2.33 [2.28-2.39] | Obesity | 2.29 [2.25-2.34] | 2,37 [2,32-2,42] |
| Cancer | 3.77 [3.70-3.85] | 2.29 [2.25-2.33] | 2.95 [2.89-3.00] | 2.39 [2.34-2.43] | Cancer | 3.33 [3.28-3.37] | 2,62 [2,59-2,66] |
| Post-partum | 22.71 [19.19-26.87] | - | 18.07 [15.24-21.43] | - | Post-partum | 20.19 [17.91-22.76] | - |
| Men | 1.22 [1.20-1.24] | 1.16 [1.14-1.18] | 1.32 [1.30-1.34] | 1.14 [1.12-1.15] | Men | 1.28 [1.26-1.29] | 1,11 [1,10-1,12] |
| Age (ref=18-50) |  |  |  |  | Age (ref=18-50) |  |  |
| 51-70 | 2.79 [2.72-2.87] | 2.42 [2.36-2.48] | 2.78 [2.71-2.84] | 2.34 [2.28-2.39] | 51-70 | 2.78 [2.73-2.83] | 2,36 [2,32-2,41] |
| > 70 | 5.00 [4.87-5.12] | 4.09 [4.00-4.18] | 4.66 [4.56-4.77] | 3.72 [3.64-3.81] | > 70 | 4.82 [4.74-4.90] | 3,96 [3,90-4,03] |

OR: odds ratio ; aOR: adjusted odds ratio ; CI: confidence interval

**Additional file 1: Figure S3: monthly distribution (%) of patients hospitalized with COVID-19 or pulmonary embolism in 2020 (with COVID-19 or not) including error bars (error bars were calculated for each month but are not necessarily easily readable because the range of confidence intervals is small) and respective incidence of COVID-19 in France (per 100,000 persons) derived from official French Government figures available from May 2020**
